# Organic nitrogen uptake is a significant contributor to nitrogen economy of subtropical epiphytic bryophytes

**DOI:** 10.1038/srep30408

**Published:** 2016-07-27

**Authors:** Liang Song, Hua-Zheng Lu, Xing-Liang Xu, Su Li, Xian-Meng Shi, Xi Chen, Yi Wu, Jun-Biao Huang, Quan Chen, Shuai Liu, Chuan-Sheng Wu, Wen-Yao Liu

**Affiliations:** 1 Key Laboratory of Tropical Forest Ecology, Xishuangbanna Tropical Botanical Garden, Chinese Academy of Sciences, Kunming, Yunnan 650223, P. R. China; 2 University of Chinese Academy of Sciences, Beijing 100049, P. R. China; 3 Key Laboratory of Ecosystem Network Observation and Modelling, Institute of Geographic Sciences and Natural Resources Research, Chinese Academy of Sciences, Beijing 100101, P. R. China; 4 Ailaoshan Station for Subtropical Forest Ecosystem Studies, Jingdong 676209, P. R. China

## Abstract

Without any root contact with the soil, epiphytic bryophytes must experience and explore poor, patchy, and heterogeneous habitats; while, the nitrogen (N) uptake and use strategies of these organisms remain uncharacterized, which obscures their roles in the N cycle. To investigate the N sources, N preferences, and responses to enhanced N deposition in epiphytic bryophytes, we carried out an *in situ* manipulation experiment via the ^15^N labelling technique in an Asian cloud forest. Epiphytic bryophytes obtained more N from air deposition than from the bark, but the contribution of N from the bark was non-negligible. Glycine accounted for 28.4% to 44.5% of the total N in bryophyte tissue, which implies that organic N might serve as an important N source. Increased N deposition increased the total N uptake, but did not alter the N preference of the epiphytic bryophytes. This study provides sound evidence that epiphytic bryophytes could take up N from the bark and wet deposition in both organic and inorganic N forms. It is thus important to consider organic N and bark N sources, which were usually neglected, when estimating the role of epiphytic bryophytes in N cycling and the impacts of N deposition on epiphytic bryophytes in cloud forests.

Bryophytes are the earliest land plants^1^,^2^,^3^, they have experienced nearly 450 million years of evolution, and they lack the supracellular transport systems of vascular plants^4^,^5^. In total, over 20,000 bryophyte species have been recorded worldwide, making them the second most diverse group of plants^6^. Bryophytes occur in many ecosystems, from low to high latitudes and altitudes, and generally dominate montane, boreal, and arctic ecosystems where they can strongly influence the carbon and nitrogen (N) cycles^7^,^8^,^9^. Unlike vascular plants, bryophytes lack a cuticle barrier and there is the existence of large cationic exchange properties within the cell walls. They can therefore take up water and nutrients over the entire plant surface^9^,^10^. Thus, these organisms often serve as effective traps for environmental nutrients, such as N, which makes them very sensitive to atmospheric N deposition, and in recent years they have been proposed to be good bio-indicators for N pollution^9^,^11^,^12^. Bryophytes can also regulate the ecosystem N dynamic through biological N_2_ fixation by forming facultative symbioses with diazotrophs, such as *Nostoc* spp.^7^,^13^,^14^. However, the details of the N preferences and the N sources of bryophytes have been less of a concern, and they remain uncharacterized in natural ecosystems^15^, which prevents a proper evaluation of their roles in the N cycle and a reasonable prediction of their fates in a changing world.

Most higher plants primarily derive N from the soil^16^. In contrast, bryophytes lack roots and developed vascular systems, which is thought to limit their access to available nutrients from substrata and affect N transport to the shoots. Previous studies have suggested that bryophytes obtain most of their nutrients from the atmospheric deposition and throughfall^17^,^18^ and atmospheric N_2_ fixation through epiphytic cyanobacteria^7^,^19^; however, this is under debate because recent evidence suggests that bryophytes may use a certain amount of N from their substrata^15^,^20^,^21^. For example, Ayres, *et al*.^21^ reported that some bryophyte species can obtain N directly from the soil. In addition to terrestrial species, many bryophytes grow on other plants as epiphytes in the canopy habitats in montane and subalpine forests^8^,^22^,^23^. The canopy habitats are usually considered to be harsh, with a variable and sporadic nutrient input, a limited storage capacity for available water and nutrients, and low physical stability, *etc*.^24^,^25^. Accordingly, epiphytic bryophytes must experience and explore poor, patchy, and heterogeneous environments. It remains unknown where and how these organisms obtain nutrients without root contact with the soil.

The N preferences of epiphytic bryophytes remain uncharacterized. The preferences for ammonium (NH_4_^+^) or amino acids over nitrate (NO_3_^−^) have been observed in vascular plants when different N forms are supplied in equal doses^26^,^27^. Some researchers have suggested that bryophytes may not have a preference in N uptake because nutrients can enter moss tissues easily through cation exchange and the proton (H^+^) pump (e.g., NH_4_^+^ and amino acids) and through cotransport (e.g., NO_3_^−^) for positively charged ions^10^,^15^,^28^. Other researchers have suggested that the uptake of NH_4_^+^ should be higher than that of NO_3_^−15 ^29. Current knowledge of N cycling in bryophytes and the effects of N deposition on bryophytes largely relates to inorganic N^30^,^31^,^32^. The ability of bryophytes, especially the epiphytic ones, to use organic N as a N source and the ecological significance of this has been largely neglected in past studies, although the preference of amino acids has been reported in several terrestrial bryophytes^33^,^34^.

Anthropogenic N deposition has been increasing globally since the 19^th^ century, which has triggered major changes in the dynamics of carbon (C) and N, as well as floral diversity in terrestrial ecosystems^35^,^36^,^37^. Increasing concern has been focused on the effects of enhanced N deposition on bryophytes, which were suggested to be sensitive to N pollution^11^,^18^,^38^. A previous study indicated that species richness and the cover of the epiphytic bryophyte community has significantly decreased because of increased N input. The growth and vitality of the investigated species have declined in locations with high N loads^11^. However, we still do not know the potential impacts of increased N deposition on the total N uptake and the N preference of epiphytic bryophytes. For example, do bryophytes absorb more N in response to increased N deposition, as has been suggested in some studies^39^,^40^? Do bryophytes shift their N preference to increased N deposition when N is much more easily obtained? This information may provide possible explanations for the detrimental effects of the high N loads mentioned above.

Due to their particular biological nature (no cuticle barrier, lacking roots and developed vascular systems, and growing on bark, *etc*.) and special habitats (poor, patchy, and heterogeneous in N supply), epiphytic bryophytes are likely to have different N uptake and use strategies under natural conditions and under increasing atmospheric N deposition. Epiphytic bryophytes usually dominate the tree trunks and branches in montane forests on moist, undisturbed sites^8^,^41^. For example, the subtropical montane cloud forest located in the Ailao Mountain National Nature Reserve of Southwest China, which is generally characterized by persistent and frequent cloud cover at the canopy level, is especially rich in epiphytic bryophytes^42^. In total, 176 epiphytic bryophyte species have been recorded (accounting for ~30% of the total epiphytes in the study region)^41^ and the total biomass of the epiphytic bryophytes is 6.7 tons per hectare (accounting for ~63% of the total epiphytes in the study region)^41^,^43^. A multifactor *in situ* manipulation experiment was carried out via the ^15^N labelling technique in three coexisting and common epiphytic bryophyte species in the subtropical cloud forest. The main objectives were to: 1) Determine whether epiphytic bryophytes can take up N from tree bark or bark surface; 2) Confirm the capacity of epiphytic bryophytes to absorb organic N and quantify its amount in the total N economy of the bryophytes; 3) Address the potential impacts of increased N deposition on the N uptake dynamics of epiphytic bryophytes.

## Results and Discussion

### Direct uptake of N from bark

**Figure 1:**
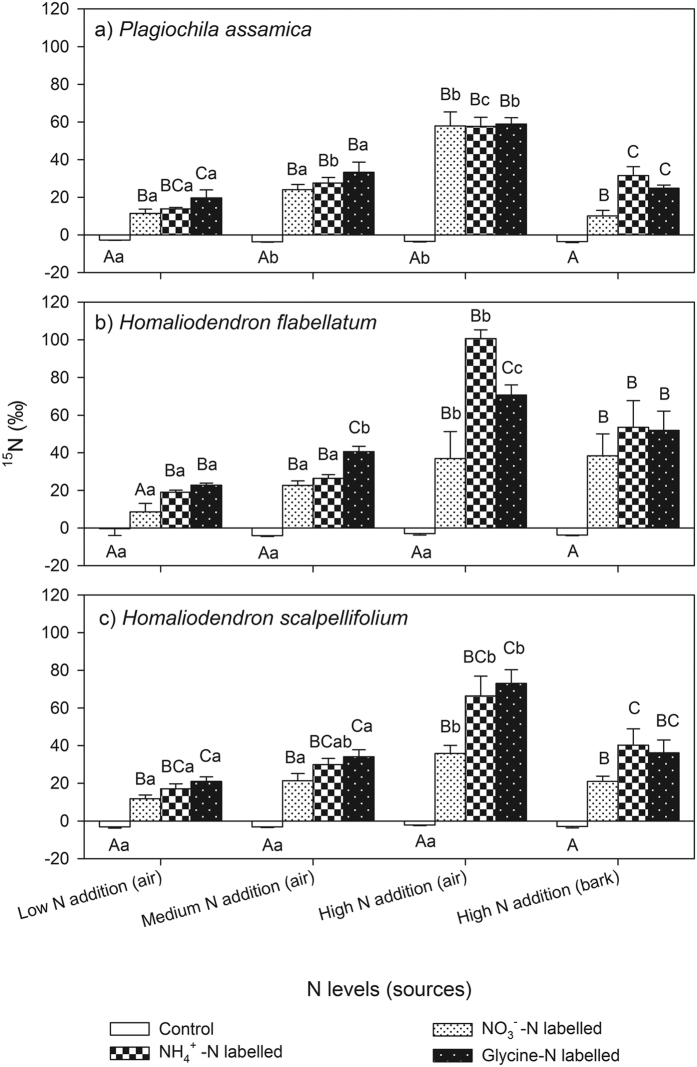
Abundance of nitrogen (N) isotope signatures (δ^15^N) in three epiphytic bryophyte species. Capital letters after values indicate significant differences at 0.05 error probability levels among different labelled-N forms under same N levels, while lowercase letters indicate significant differences among different N levels under same labelled-N forms.

Using a ^15^N labelling approach, we confirmed that epiphytic bryophytes indeed relied more on N from the air than from the bark of their hosts, but the contribution of the N from the bark should not be neglected (Tables 1 and 2; Fig. 1). Cryptogams, such as bryophytes, which can absorb water directly through their surfaces, have been traditionally suggested to be largely independent of their substrate and were thought to absorb N mostly from precipitations and biological N fixation^19^,^44^ as well as the relocation of nutrients from dead moss tissue^45^. It is surprising that epiphytic bryophytes could use the bark N because bryophytes possess rhizoids rather than roots, which reach only a few cm into the bark surface. However, the recovery of the label in the bryophyte tissue (Fig. S1) does not necessarily reflect active uptake by the bryophyte, since the added N could have reached the bryophyte via passive diffusion along the bryophyte shoots. Endophytic fungi could have retained the added label and get some amino N mineralization and uptake as NH_4_^+^. It is also possible that not all bacteria have been killed by the ampicillin once it was injected into the bark. Nevertheless, the ^15^N enrichment in the bryophyte shoots suggest that the epiphytic bryophytes can acquire N from the bark and translocate it to their shoots via various pathways, e.g. passive diffusion, endophytic fungi, bacteria. Therefore, this still need further investigations in the future.

During the injection procedure, we observed a small amount of leakage of the solution that had been injected into the bark. This was probably due to the limited water-holding capacity of the bark during the injection process. A spot of the leaked solution may flow directly onto the bryophyte shoots, and thus may have been taken up at that point, which obscure the interpretation of our results. Nevertheless, the proportion of ^15^N recovered in the trunk-dwelling bryophyte *P. assamica* after 24 h incubation was 3.2% for NO_3_^−^, 6.4% for NH_4_^+^, and 5.4% for glycine, respectively (Fig. S1). Similar results were found in the other two species, *H. flabellatum* and *H. scalpelifolium* (Fig. S1). Although lower than the air deposition counterparts (Fig. S1), the proportion of ^15^N recovered in the three bryophyte species through bark injection was comparable with previously reported data on the recovery from soil by the Antarctic moss *Sanionia uncinata* (2% for alanine and 4% for NH_4_^+^)^46^. Thus, the fact that a small amount of liquid leaked out of the bark may not significantly impact the conclusion that epiphytic bryophytes can take up N from bark.

Yet it has been demonstrated that bryophytes can take up N from bark when it is added, but is there actually a significant amount of available N in bark for bryophytes to take up? The average total N concentration in barks of the three dominant host species, *i.e. Lithocarpus xylocarpus*, *L. hancei*, and *Castanopsis wattii* are 7.85 ± 0.61, 8.92 ± 1.33, 8.52 ± 0.49 g kg^−1^, respectively, which are higher than the average total N concentration in the surface soil (0–20 cm: 6.53 ± 0.83 g kg^−1^) (Song *et al*. unpublished data). Mean concentrations of total N, NH_4_^+^–N, and NO_3_^−^–N in the stemflow in the study region are 2.39 ± 1.11, 1.14 ± 0.50, and 0.42 ± 0.21 mg l^−1^, respectively, which are significantly higher than that in the precipitation (0.49 ± 0.14, 0.11 ± 0.05, 0.04 ± 0.01 mg l^−1^)^47^. The above data indicate that there is actually a significant amount of available N in bark for epiphytic bryophytes to take up, which is comparable to N availability from atmospheric deposition and other substrates in the subtropical cloud forest.

Field investigations have indicated that bryophytes are the dominant epiphytic plants in this ecosystem, as they account for approximately 63% of the total biomass, which is more than any other epiphytic vegetation type (orchids, ferns, and lichens, *etc*.)^41^. This indicates that epiphytic bryophytes may be significant competitors for N in the stemflow and throughfall, which could have potential consequences for the plant community structure and nutrient cycling at the ecosystem level^47^,^48^. Since all three bryophyte species included in this study have the capacity to absorb the available N from the bark, according to the δ^15^N signals of the shoots (Fig. 1), this uptake may be common among epiphytic bryophyte species in general. Increasing evidence indicates that both the substratum and the atmosphere are important mineral sources for bryophytes, even in ‘feather mosses’, which have poor soil-moss contact^46^,^49^. Recently, Liu, *et al*.^15^ estimated that soil N accounted for approximately 40% of the total N in terrestrial bryophytes. If N absorption from bark is common among epiphytic bryophytes, this could partially explain their widespread distribution and importance in many montane and moist ecosystems. If this is the case, the N cycle in the studied forest ecosystem should be modified.

### Organic N as important component of N input

**Figure 2:**
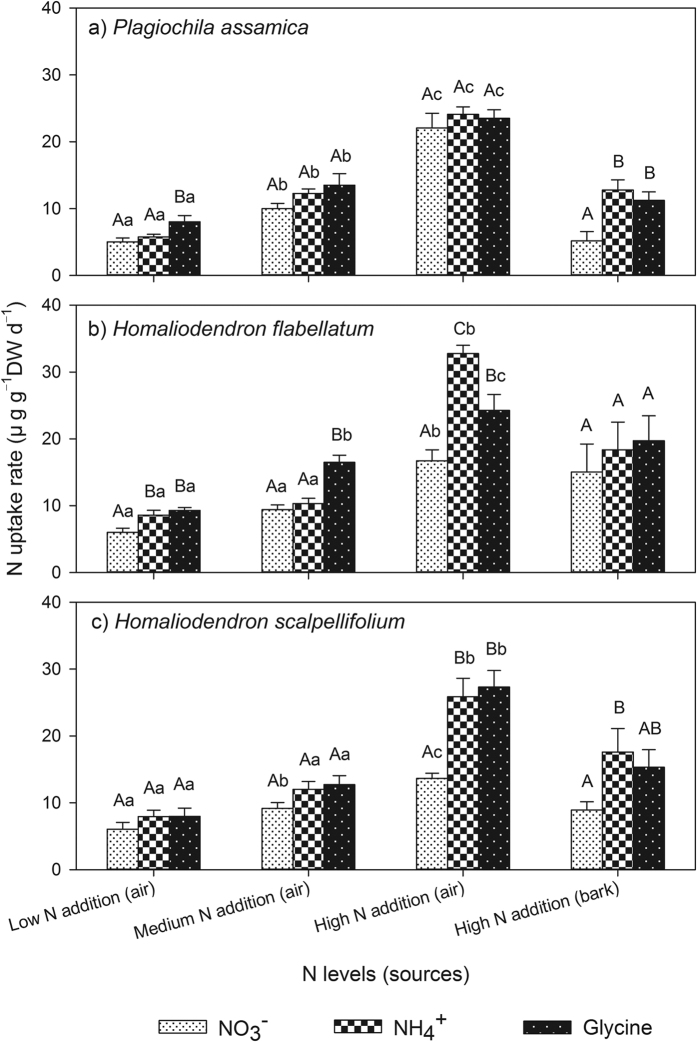
Comparisons of nitrogen (N) uptake rates by three bryophyte species from NO_3_^−^, NH_4_^+^, and glycine under air deposition and bark injection at low, medium, and high N addition levels. Capital letters after values indicate significant differences at 0.05 error probability levels among different labelled-N forms under same N levels, while lowercase letters indicate significant differences among different N levels under same labelled-N forms.

Bryophytes have the ability to absorb organic N besides mineralized, inorganic N (NH_4_^+^ and NO_3_^−^). In this study, the contribution of organic N (glycine) ranged between 28.4% and 44.5% of the total N uptake, which was comparable with NH_4_^+^, but significantly higher than NO_3_^−^ (Tables 1 and 2; Fig. 2). This was probably due to the greater cation-exchange capacity than the anion-exchange capacity of the cell walls^29^,^50^. No significant differences were detected for the acquisition of different N forms between air deposition and bark injection for all three epiphytic bryophyte species (Table 2), which demonstrated that these organisms do not shift their N preference from air to bark N sources. Our study indicated that amino acids, a small but important organic N component, as well as NH_4_^+^–N and NO_3_^−^–N can serve as important N sources for epiphytic bryophytes. Previous laboratory and field studies have also revealed that amino acids can be absorbed and utilized at substantial rates, which greatly contribute to the total N uptake and effects the N metabolism of bryophytes^15^,^34^,^51^,^52^; however, this has not been studied in epiphytic bryophyte species. For example, Forsum, *et al*.^33^ applied ^15^N labelled solutions to *Hylocomium splendens* (Hedw.) and found that this species preferred amino acid N over NO_3_^−^, although the assimilation of glycine remained lower than that of NH_4_^+^. The mean uptake rates were 1.8 μmol g^−1^ DW h^−1^ for NO_3_^−^, 3.6 μmol g^−1^ DW h^−1^ for NH_4_^+^, and 3.4 μmol g^−1^ DW h^−1^ for glycine, which indicated that the amino acids could be absorbed by the bryophytes^50^. The preference for amino acid N or NH_4_^+^ over NO_3_^−^ observed could be partially explained by their differences in assimilation costs. According to Liu, *et al*.^15^, and references therein, the assimilation cost of amino acids is expected to be lower than that of NH_4_^+^ and much lower than that of NO_3_^−^, which is due to the requirement that NH_4_^+^ must be attached to a C skeleton before use while NO_3_^−^ requires additional reduction steps to NH_4_^+^. These studies demonstrated that amino acids should be one of the most cost-effective N forms that can be utilized by bryophytes. Considering that amino acids account for only a small proportion of organic N, the bioavailable fraction of organic N is expected to be much larger than that found in amino acids^53^. Although the organic N input in the studied forest has never been directly measured, the fact that the annual input of total N through precipitation (10.5 kg N ha^−1^ y^−1^) was *ca*. threefold of the sum of the two main inorganic-N forms (NO_3_^−^: 0.91 kg N ha^−1^ y^−1^, NH_4_^+^: 2.69 kg N ha^−1^ y^−1^)^47^, implies that organic N may be an important N form in the studied region. The contribution of organic N to the N economy of epiphytic bryophytes might have been seriously underestimated in the past, although uncertainty exists considering some organic N, *e.g*. amino acids may have been mineralized and been took up as NH_4_^+^.

### Impact of enhanced N deposition on N uptake

In this study, the shoot ^15^N concentration and the N uptake rates increased significantly with increasing N concentrations (Table 1; Figs 1 and 2), but the N preferences of the three bryophyte species shifted only slightly in response to the addition of N, except for *Homaliodendron flabellatum* that preferred glycine under medium N addition, but shifted to NH_4_^+^ under high N addition (Table 2). This implies that increased N deposition increases the total amount of N absorbed by the epiphytic bryophytes, but it does not alter the N preference over a short time. Due to N limitations in many ecosystems dominated by bryophytes, a slight increase in N can increase the absorption of N, which enhances the chlorophyll content of the bryophyte cells, thus increasing the photosynthetic capacity^54^,^55^. However, excessive N supply is detrimental to these sensitive organisms. For example, it has been demonstrated that the oversupply of N can result in an excess uptake of NH_4_^+^ in the cell, which threatens the cell homeostasis and causes toxicity, and thus a subsequent reduction in the growth of the bryophytes^55^,^56^. Increased N deposition can alternatively cause increased amino acid accumulation in bryophyte tissues, which may deplete reserves of soluble carbohydrates necessary for growth^54^. As indicated in this study, high N loads resulted in excessive N uptake, which may induce biochemical disorders in bryophytes^57^.

In conclusion, this study provides clear evidence that epiphytic bryophytes can uptake N from the bark and can translocate it to their shoots. The ability to translocate the absorbed N to their shoots is of particular importance, since shoots typically have greater N demands for photosynthetic enzymes. This study highlighted that organic N, as opposed to inorganic N, contributed remarkably to the N economy of the epiphytic bryophytes. High N loads may result in excessive N uptake, which may induce biochemical disorders in bryophytes. Thus, it is important to consider organic N and bark N sources when estimating the role of epiphytic bryophytes in N cycling and the impacts of N deposition on epiphytic bryophytes in cloud forests.

## Methods

### Study site

We conducted this study in the Xujiaba region of Yunnan Province (24° 32′ N, 101° 01′ E), China, in a protected section of a 5,100 ha pristine subtropical cloud forest in the Ailao Mountain National Nature Reserve (23° 35′-24° 44′ N, 100° 54′-101° 01′ E), with an altitude range between 2000 m and 2600 m^58^. The mean annual temperature is 11.6 °C, with the lowest value in December (6.0 °C) and the highest in July (15.8 °C). The mean annual rainfall is 1859 mm, with 86% of the rain falling during the rainy season (May to October), and a pronounced dry period from December to April^42^. The forest is primarily co-dominated by *Lithocarpus hancei* (Benth.) Rehder, *Castanopsis rufescens* (Hook.f.et Th.) Huang et Y.T. Chang, and *Lithocarpus xylocarpus* (Kurz) Markgr^58^. Annual input of total N through precipitation and throughfall were 10.5 and 12.1 kg N ha^−1^ y^−1 ^47 in the study region, with expectations of increased reactive N deposition with time^59^.

On account of the persistent, frequent cloud/fog cover, the presence of large, old trees, and long-term effective protection, the forest harbors abundant epiphytes, and is especially rich in epiphytic bryophytes^41^,^42^. The most dominant epiphytes growing on tree trunks including *Homaliodendron flabellatum* (Sm.) Fleisch., *Plagiochila arbuscula* (Brid. ex Lehm.) Lindenb., *H. scalpelifolium* (Mitt.) Fleisch., and *P. assamica* Steph. are bryophyte species^41^. Epiphytes comprise one of the most diverse and are a conspicuous element of the subtropical cloud forest, and they are also extremely important in carbon, water and nutrient cycling in these ecosystems^47^,^48^. For example, epiphytes in the subtropical cloud forest can fix a significant amount of N_2_; the latest estimated annual N input fixed by epiphytic bryophytes reaches 3.89 kg N ha^−1^ y^−1 ^60,61.

### Experimental design and treatments

Two mosses (*H. flabellatum* and *H. scalpellifolium*) belonging to Neckeraceae and one liverworts (*P. assamica*) belonging to Plagiochilaceae were selected for this manipulation experiment as they were abundant, representative trunk-dwelling species in the study region^41^,^42^. In November 2014, four areas (two hectares each) in the cloud forest in Xujiaba were chosen as the experimental plots. In each plot, 48 square quadrats (20 cm × 20 cm) that were dominated by each single species (16 quadrats for each species) were marked on large trunks (Diameter at breast height >20 cm) that were located between 1.0 m–2.0 m above the forest floor. The average total biomass of *H. flabellatum*, *H. scalpellifolium*, and *P. assamica* collected from each experimental quadrat were 1.01, 1.13, and 1.46 g, respectively (Fig. S2).

To study the N preference of the three epiphytic bryophytes, four treatment groups were established. In each treatment, equal proportions of glycine, NH_4_^+^, and NO_3_^−^ (1:1:1) were used. Glycine was adopted because it has been widely used as a model amino acid for studies of organic N uptake by plants^62^. The first treatment was considered to be the control (Control), and no ^15^N-labelled N was added. In the other three treatments, only one of the N forms was labelled with ^15^N, i.e., ^15^N–glycine (20 atom% ^15^N) mixed with unlabelled (NH_4_)_2_SO_4_ and KNO_3_ (Glycine–N labelled); (^15^NH_4_)_2_SO_4_ (20 atom% ^15^N) mixed with unlabelled glycine and KNO_3_ (NH_4_^+^–N labelled); and K^15^NO_3_ (20 atom% ^15^N) mixed with unlabelled glycine and (NH_4_)_2_SO_4_ (NO_3_^−^–N labelled).

The experiment was divided into two parts. First, we aimed to determine if epiphytic bryophytes could absorb the available N directly from the tree bark. For this, a subset of high N solutions (total N concentration of 12 mM: each N form at 4 mM) with the four treatment groups mentioned above (CK, Glycine-N labelled, NH_4_^+^–N labelled, NO_3_^−^–N labelled) were injected uniformly into the bark of the trees in the 48 marked quadrats (four N forms × three species × four replicates) with 5 mL syringes. Before the injection process, a steel needle (15 cm in length and 3.5 mm in diameter) was used to squeeze through the bryophyte layer and set up injection holes on barks. The trees were then injected at nine injection points (three rows × three columns) each on the bark of all quadrats to approximately a 5 mm depth using special metal frame sheets (20 cm × 20 cm) to ensure uniformity and consistency. The total amount of N injected to each quadrat corresponded to a dose of 0.21 kg N ha^−1^.

The second experiment was designed to study the treatment effect of increased N addition on the N preference and uptake. Three N levels, i.e., low (total N concentration 3 mM: each N form at 1 mM), medium (6 mM: each N form at 2 mM), and high (12 mM: each N form at 4 mM), of each treatment were added. In this experiment, 5 ml of the experimental solutions with different N concentrations were sprayed uniformly over 144 experimental quadrats (three N levels × four N forms × three species × four replicates) with small pressure sprayers. The total amounts of N added were 0.05, 0.11, and 0.21 kg N ha^−1^ (equal to 18.25, 40.15, 76.65 kg N ha^−1^ y^−1^) for the low, medium, and high N levels, respectively. The above treatment levels in our simulations of N input were implemented based on background rate (10.5 kg N ha^−1^ y^−1^) in the study region^47^ and a predicted rate measured in a comparable region, i.e. southern China: 30–73 kg N ha^−1^ y^−1 ^63. Spraying was conducted within special metal frame cubes (20 cm × 20 cm × 20 cm) to avoid the loss of the solutions through air movement. Ampicillin (10 mg L^−1^) and CaCl_2_ (100 μM) were added into the solutions to avoid the rapid decomposition of the amino glycine. Bryophyte shoots were harvested one day after the injection or spraying events, following the procedures of Krab, *et al*.^51^ and Rousk, *et al*.^46^. According to the method used by Warren^64^, all bryophyte shoots were rinsed in 50 mM KCl to remove any remaining ^15^N that was still adhering to their surface, and then they were rinsed with ultrapure water, oven-dried (70 °C), and ground for ^15^N isotope analysis. The total biomasses of the three target bryophyte species are shown in Fig. S2. N contents and ^15^N/^14^N ratios were determined using an isotope ratio mass spectrometer (Isoprime 100, Isoprime Ltd., Cheadle, UK) coupled with a vario PYRO cube elemental analyzer (Elementar Analysensysteme GmbH, Hanau, Germany) with a continuous flow mode. The atom% excess ^15^N (APE) was calculated as the atom% ^15^N difference between the bryophytes from the ^15^N treated plots and from the control plots.

### Data analysis

According to Xu, *et al*.^65^, the uptake of the ^15^N (mg ^15^N m^−2^) by the bryophyte shoots was calculated by multiplying the N content (mg N g^−1^ d.w.), APE, and biomass (g m^−2^). The uptake of the available N forms corresponding to the ^15^N treatment was calculated as in the following:


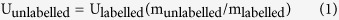


where m_labelled_ is the total mass (g m^−2^) of the ^15^N-labelled N injected or sprayed per quadrat and m_unlabelled_ is the mass of available N forms measured in solution. U_labelled_ is the uptake (g m^−2^) of ^15^N from the source m_labelled_ and U_unlabelled_ is the uptake of the available N from the source m_unlabelled_.

All data were subjected to normality and homoscedasticity tests before statistical analysis. Two different ANOVA models were adopted: one focused on the N source and the other focused on the N level. To compare N sources, only the high N treatment groups through both air spraying and bark injecting were compared. To compare the treatment effects of the N levels, only the air spraying treatment groups, including low, medium, and high N levels, were considered.

Multiple comparisons of the shoot δ^15^N, N uptake rates, and contributions of different N forms (percentages of N absorbed in the forms of NO_3_^−^, NH_4_^+^, and glycine) among the labelled-N forms under the same N addition levels and among different N addition levels under the same labelled-N forms were conducted using LSD’s or Game-Howell’s post hoc tests. All the analyses mentioned above were conducted in SPSS 16.0 (SPSS, Chicago, IL, USA), and all figures were made using SigmaPlot 12.5 (Systat Software Inc., San Jose, CA, USA).

## Additional Information

**How to cite this article**: Song, L. *et al*. Organic nitrogen uptake is a significant contributor to nitrogen economy of subtropical epiphytic bryophytes. *Sci. Rep.* **6**, 30408; doi: 10.1038/srep30408 (2016).

## Supplementary Material

Supplementary Information

## Figures and Tables

**Table 1:** Results from an ANOVA analysis evaluating the effect of species, nitrogen (N) sources, N forms, N levels, and their interactive effects on N uptake rates during treatment periods. Italics indicates *p* < 0.05.

	**DF**	**MS**	***F*****-value**	***p*****-value**
Species (S)	2	134.85	5.676	*0.006*
N sources (N_s_)	1	1648.107	69.371	<*0.001*
N forms (N_f_)	2	463.654	19.516	<*0.001*
S × N_s_	2	72.625	3.057	0.055
S × N_f_	4	29.531	1.243	0.304
N_s_ × N_f_	2	19.09	0.804	0.453
S × N_s_ × N_f_	4	56.872	2.394	0.062
Species (S)	2	0.262	2.309	0.106
N levels (N_l_)	2	42.254	371.933	<*0.001*
N forms (N_f_)	2	4.805	42.294	<*0.001*
S × N_l_	4	0.137	1.209	0.314
S × N_f_	4	0.274	2.41	0.056
N_l_ × N_f_	4	0.633	5.574	*0.001*
S × N_l_ × N_f_	8	0.411	3.622	*0.001*

DF refers to degree of freedom and MS refers to mean square.

**Table 2:** Percentages of nitrogen (N) absorbed in forms of NO_3_^−^, NH_4_^+^, and glycine by three bryophyte species under air deposition and bark injection at low, medium, and high N addition levels.

**Species**	**Low N addition (air)**			**Medium N addition (air)**			**High N addition (air)**			**High N addition (bark)**		
	**NO**_**3**_^**−**^	**NH**_**4**_^**+**^	**Glycine**	**NO**_**3**_^**−**^	**NH**_**4**_^**+**^	**Glycine**	**NO**_**3**_^**−**^	**NH**_**4**_^**+**^	**Glycine**	**NO**_**3**_^**−**^	**NH**_**4**_^**+**^	**Glycine**
*Plagiochila assamica*	25.3 ± 3.3	36.5 ± 0.6	38.2 ± 3.0	26.6 ± 5.9	41.3 ± 3.9	32.1 ± 2.4	23.1 ± 2.2	43.7 ± 4.8	33.2 ± 3.2	21.1 ± 5.0	40.3 ± 4.6	38.6 ± 6.4
	Aa	Ba	Ba	Aa	Ba	ABa	Aa	Ba	ABa	A	B	B
*Homaliodendronflabellatum*	21.0 ± 2.7	39.7 ± 2.9	39.3 ± 5.4	27.7 ± 4.6	27.7 ± 1.7	44.5 ± 3.6	21.2 ± 4.3	50.4 ± 5.0	28.4 ± 4.1	27.7 ± 6.9	40.0 ± 8.0	32.3 ± 5.1
	Aa	Bab	Bab	Aa	Ab	Ba	Aa	Ba	Ab	A	A	A
*Homaliodendronscalpellifolium*	23.1 ± 3.2	40.6 ± 3.1	36.3 ± 4.3	23.3 ± 3.1	39.1 ± 5.5	37.5 ± 6.4	20.0 ± 3.2	31.5 ± 4.4	48.5 ± 7.5	20.5 ± 2.5	41.4 ± 6.0	38.1 ± 3.6
	Aa	Ba	Ba	Aa	Aa	Aa	Aa	ABa	Ba	A	B	B

Means ± SE are presented (n = 4). Capital letters after values indicate significant differences at 0.05 error probability levels among different labelled-N forms under same N levels, while lowercase letters indicate significant differences among different N levels under same labelled-N forms.
