# Supplementary material for: Organic nitrogen uptake is a significant contributor to nitrogen economy of subtropical epiphytic bryophytes
Source: Sci Rep. 2016 Jul 27;6:30408. doi: 10.1038/srep30408 (PMC4961951; doi:10.1038/srep30408)

Supplementary Information:

Liang Song, Hua-Zheng Lu, Xing-Liang Xu, Su Li, Xian-Meng Shi, Xi Chen, Yi-Wu, Jun-Biao Huang, Quan Chen, Shuai Liu, Chuan-Sheng Wu, Wen-Yao Liu. Organic nitrogen uptake is a significant contributor to nitrogen economy of subtropical epiphytic bryophytes.

Fig. S1. Comparisons of ^{15}N recovery by three bryophyte species from NO_3^-, NH_4^+, and glycine under air deposition and bark injection at low, medium, and high N addition levels.

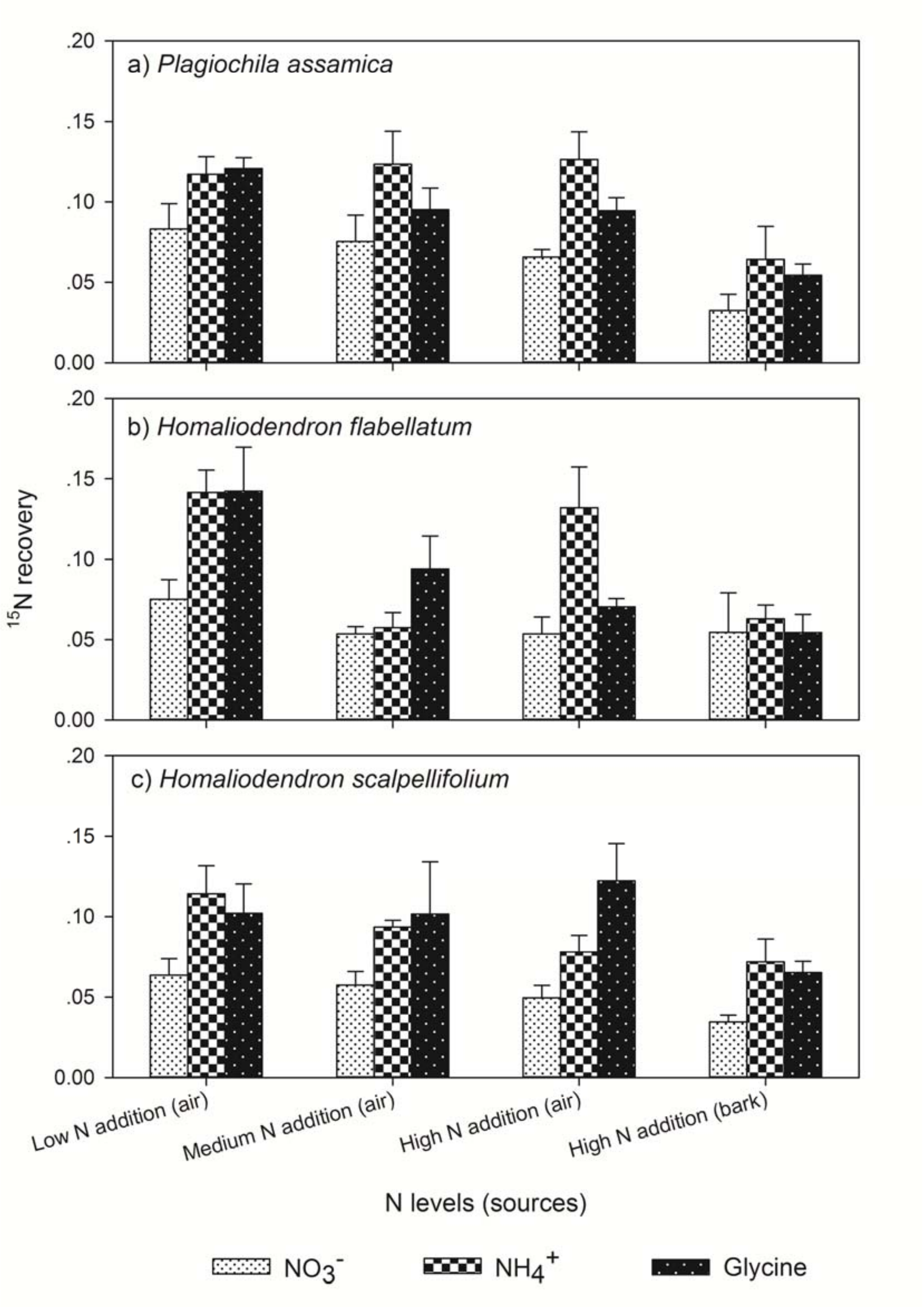

Fig. S2. Total bryophyte biomass (g) in the experimental quadrats (20 cm × 20 cm) that dominated by each single species of *Homaliodendron flabellatum* (Sm.) Fleisch., *H. scalpellifolium* (Mitt.) Fleisch., and *Plagiochila assamica* Steph. Values are means (± SE) of 16 replications.

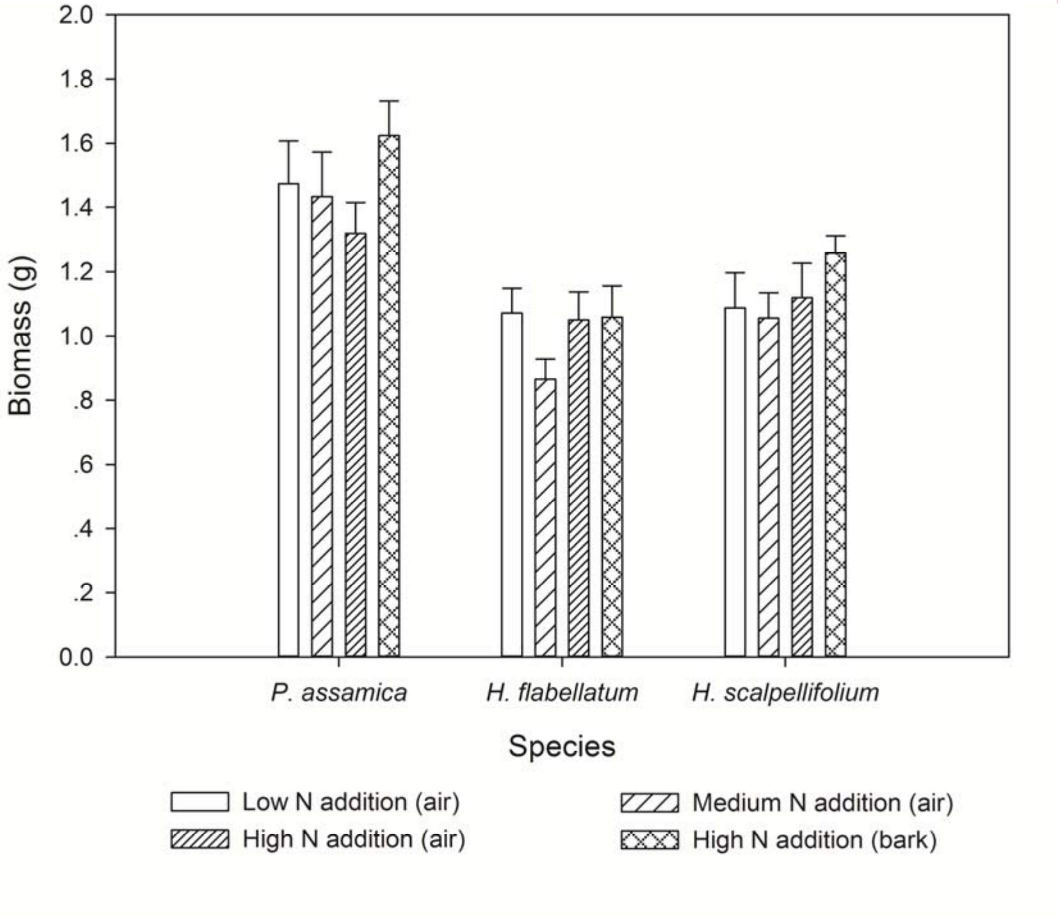

Supplement: Supplementary Information [file srep30408-s1.pdf]
